# Improving Breast Cancer Outcomes among Women in China: Practices, Knowledge, and Attitudes Related to Breast Cancer Screening

**DOI:** 10.1155/2012/921607

**Published:** 2012-12-09

**Authors:** Tsu-Yin Wu, Yi-Lan Liu, Scott Chung

**Affiliations:** ^1^School of Nursing, Eastern Michigan University, 311 Everett L. Marshall Building, Ypsilanti, MI 48197, USA; ^2^Wuhan Union Hospital, Tongji Medical College and Department of Nursing, Huazhong University of Science and Technology, Wuhan 430022, China; ^3^The College of Literature, Science, and the Arts, University of Michigan, Ann Arbor, MI 48109, USA

## Abstract

*Background*. Breast cancer is a major public health issue and the most commonly diagnosed cancer for women worldwide. Despite lower incidence rates than those living in Western countries, breast cancer incidence among Chinese women has increased dramatically in the past 20 years. Nevertheless, there is a paucity of studies reporting the attitudes toward and practices of breast cancer screening among Chinese women. *Methods*. This cross-sectional study examined the practices, knowledge, and attitudes toward breast cancer screening (BCS) on a convenience sample of 400 Chinese women. *Results*. Among study participants, 75% of the women never had a mammogram and the top three barriers reported were low priority, feeling OK, and lack of awareness/knowledge toward breast cancer screening. The results from the logistic regression model showed increased self-efficacy; having performed monthly self-exams, and having had clinical breast exams in the past two years were significant correlates while demographic variables were not correlated with screening behaviors. *Conclusion*. The findings provide a foundation to better understand beliefs and practices of Chinese women toward BCS and highlight the critical need for general public, health professionals, and the health care system to work collaboratively toward improving the quality of breast cancer care in this population.

## 1. Introduction

Breast cancer is a major public health issue and the most commonly diagnosed cancer for women worldwide [1–3]. Despite the lower incidence rate compared to those living in Western countries, breast cancer incidence in Chinese women has increased dramatically in the past 20 years, with a total increase of 50–100% [4]. During the 1970s, the average age-standardized incidence rate of breast cancer for Chinese women living in Shanghai was about 18.3 per 100,000 women and by 1989, the age-standardized incidence rate for breast cancer has increased to about 25.1 per 100,000 women [5, 6]. The mortality of female breast cancer has constantly increased in China since 1973, and the mortality in urban areas were higher than that of rural areas [7].

A recent study conducted by Ziegler and colleagues (2008) projects that based on a representative sample of Chinese women from both urban and rural areas, the age-standardized breast cancer incidence rate in China will be 87.8 per 100,000 women by the year 2021 [8]. This projection is corroborated by another study, which used data from the Chinese National Family Planning and Reproductive Health Survey (NFPRHS). The study estimates that the age-standardized breast cancer incidence rate in China will be 85.3 per 100,000 women in year 2021; the increase in incidence and prevalence of breast cancer is not limited to major cities in China. It is pervasive throughout all of China and has been projected to continue to increase in the future [9].

Not only is the overall incidence rate for breast cancer rising in China, but also, another intriguing fact is that women in China are diagnosed with breast cancer at an earlier age than Caucasians. In Hong Kong, the incidence of breast cancer occurs at the highest rate at the age of 40 years [10] while for Caucasians the incidence of breast cancer occurs at the highest rate between the ages of 45 years and 55 years [10]. Furthermore, the earlier diagnosis of breast cancer for Chinese women is coupled with growing evidence that breast cancer in younger women is biologically more aggressive than in their older counterparts [11–13]. Limited evidence indicates that there is a lower survival rate if breast cancer is detected at an earlier age [14, 15]. There is also evidence that suggests that age is a significant factor for relapse of breast cancer and overall mortality, whereas a young age at diagnosis is an adverse prognostic factor [15, 16]. Therefore, not only are Chinese women on average diagnosed earlier in their lives, but also the breast cancer is often more detrimental. Although it is unclear whether the disparity in incidence of breast cancer between the Chinese and Western populations is due to genetic susceptibility, evidence indicates that a “Westernized” lifestyle, which is usually defined by menarche, decreased parity, delayed childbearing, a diet rich in saturated fat, and a sedentary lifestyle, is associated with the increased incidence of breast cancer [17, 18].

Early detection of breast cancer through regular screening modalities and enhanced treatment has been found to decrease mortality rates by 25–30% [19, 20]. During the past 40 years, randomized clinical trials have demonstrated substantially reduced mortality rates from breast cancer among women who participate in screening mammography [20, 21]. Nevertheless, routine mammography screening programs commonly available in the U.S. and other Western societies have not been utilized in China. Most Chinese women are screened voluntarily or seek medical advice when an abnormality is present [12]. With the recent health care reform in China, about 60% of individuals in 2008 were covered by the three major health insurance programs, New Rural Cooperative Medical Scheme (NCMS), Urban Employees Basic Medical Insurance (UR-BMI), and Urban Residents Basic Medical Insurance for the unemployed (UR-BMI). In urban areas where the medical condition and insurance are well established, screening for breast cancer is covered by medical insurance. In rural areas, a program issued in 2009 aims to provide free breast cancer screening for all the women in 200 counties [22]. Despite the continuous effort and increasing interests among Chinese public health officials and various nonprofit organizations such as Cancer Foundation of China and Chinese Anti-Cancer Association, there are currently no standard breast cancer screening guidelines, and nationwide breast cancer screening has not been implemented routinely in China. While breast cancer screening is widely accepted globally to achieve early detection of cancer and reduce mortality by downstaging the disease, there is a paucity of studies reporting the attitudes and practices of breast cancer screening among Chinese women. 

The purpose of this descriptive study was to explore the relationships of sociodemographic characteristics, knowledge, and beliefs about breast cancer (BC) and their practices to the self-reported practice of BC screening (BCS) among women in China. The specific aims were to (a) describe the sociodemographic characteristics, knowledge, beliefs, and mammography screening practices of women in China ages 40 and older and (b) identify correlates of BCS practices.

## 2. Methods

### 2.1. Setting and Procedure

In this cross-sectional study, the data was collected from the Wuhan, the capital city of Hubei Province, an important central city as the political, economic, scientific and technological, cultural, and financial center in inland China. The study sample consisted of 400 Chinese women aged 40 years and older. This study protocol was reviewed and approved by the Institutional Review Board for conducting this research.

The study participants were recruited at the gatherings of community centers, parks, and temples. The study aim was explained to eligible women, who were assured that their participation would be voluntary and confidential. After consents were obtained, the participants completed the survey, which took approximately 20–30 minutes.

### 2.2. Measurement

The study measurement survey included two parts: (a) Chinese Mammogram Screening Beliefs Questionnaire (CMSBQ) and three subscales were used in current study: (1) perceived self-efficacy (2 items; sample item: I am confident in my ability to obtain breast cancer screening regularly), (2) perceived benefits subscale (7 items; sample item: having breast cancer screening will help to find breast lumps early), and (3) perceived barriers subscale (21 items; sample item: I feel uncomfortable taking off clothes in front of health professionals during the screening). Possible responses to the items ranged from 1 (strongly agree) to 4 (strongly disagree). Each subscale was scored by calculating the means of all item scores. The findings of benefits and barriers subscales among Asian women demonstrated excellent supportive psychometric properties with promising Cronbach's alphas above 0.70, and the results from the confirmatory factor analysis supported construct validity with good model fit indices [23, 24]. The survey instrument also included demographic information (e.g., age, marital status, education, income level, and health insurance coverage). (b) The open-ended questions asked participants to identify the three most frequent barriers that they encountered and that prevented them from getting breast cancer screening, and the facilitators that would make them more likely to get screened. The participants were instructed to leave the space blank if they did not believe they had a significant barrier to using mammography. 

### 2.3. Data Analyses

Data analyses were performed using the SPSS statistical software package (Version 18.0). Descriptive statistics, including percentages, means, and standard deviation (SDs), were calculated. Next, multivariate logistic regression analysis was performed to examine the associations of demographics, knowledge, beliefs, and behavioral factors with the probability of having received mammograms/ultrasounds after accounting for all other factors in the model. These associations were expressed in the form of adjusted odds ratios with 95% confidence intervals (CI).

Qualitative analytic techniques were employed for the analysis of the open-ended questions. The primary researcher and two research associates reviewed and coded participants' responses separately to uncover emerging themes and categories of the responses. The coding of the responses was then discussed and verified by two senior investigators with expertise in cancer control and qualitative research. Once consensus on coding was reached by the research team, the set of themes/categories were finalized. To complete each content area, quotes from the responses have been highlighted to illustrate the category. Discrepancies between the two researchers were resolved through iterative discussions until consensus was reached. Both the content and frequency of the responses were then analyzed.

## 3. Results

### 3.1. Demographic Characteristics

 The sociodemographic characteristics of the study participants are presented in Table 1. The mean age of 400 participants was 51.8 years (ranging from 40 to 81 year, SD = 7.7). Ninety-six percent of the respondents were currently married. While the education levels were widely distributed, 44% of the women reported their highest education was high school, 14% with college or higher education, and 4% with no formal education. Of those who reported their monthly income, 44% reported median income levels (equals to U.S. $410–$725) while almost 1/5 of the participants reported having incomes of less than $410 (U.S. dollars) per month. The occupations of these women were more evenly distributed with 23% working blue-collar jobs and 29% working white-collar jobs. Only 2% of the respondents had a family history of breast cancer. More than half of the Chinese women (53%) respondents did not have insurance. 

### 3.2. Breast Cancer Screening: Past Practices, Intention, and Beliefs

 As shown in Table 2, despite the fact that 80% of the participants have heard of breast self-exams, only 20% of the women knew the appropriate time intervals to conduct monthly breast self-exams. Although 41% of the participants have heard of mammograms, 75% of the women never had a mammogram during the past five years; only 13% reported ever having a mammogram done in the same time interval (Table 2). Chinese women are known to have smaller and denser breasts than Western women, and therefore it is a common practice to have an ultrasound done in this country [25]. About 23% of the participants reported having ultrasounds within the span of the past five years. About 33% of women reported that they had had their breasts checked by the doctor and/or other health professionals (i.e., clinical breast exam) in the past two years. In terms of planning to be screened in the future for a mammogram or an ultrasound, less than 1/5 of the participants reported such an intention. The mean/standard deviation for the self-efficacy, benefits and barriers subscales can be found in Table 2.

### 3.3. Barriers toward Breast Cancer Screening

The study tool asked the respondents to name up to three of the most important reasons that prevented women from having regular breast cancer screening. In this sample, 346 women (86.5%) gave 807 responses related to their perceptions on barriers toward BC screening.

Ten themes emerged from the participants' responses to the question of barriers to breast cancer screening (Table 3). If a woman had multiple responses that corresponded to the same theme, they were counted only once.

As shown in Table 3, the most frequently reported barrier related to the theme of low priority (63%), participants mentioned “no time,” “too much trouble getting a screening,” “took too much time,” “lazy,” and another quote was “don't care about it.” More than half of participants (58%) of the valid responses indicated barriers that could be classified as “Don't need it because I feel OK,” and the responses included “no symptoms,” “I am healthy,” “never got any breast disease before,” “don't feel abnormal,” “don't need it as there is no breast pain,” and “don't feel lumps and abnormality.” Two responses, “I think I have low probability of getting breast cancer” and “I'm lucky and couldn't get it,” provided vivid responses to show what respondents have in mind about the cues/triggers for obtaining breast cancer screening. The third most frequently reported barrier related to lack of awareness (*n* = 145, 42%), and examples of responses included “awareness is not emphasized/enough,” “don't have related knowledge (on breast cancer or screening),” “don't know how to do screening,” and so forth. Among these responses, 22 (6%) were simply stated, “I'm too old to get screened.” About 33% of responses were related to the cost of having a mammogram; several women indicated that after retirement, they do not have insurance arrangements getting mammograms. Less than 10% of the valid responses could be classified as “logistics,” including “do not know where to go for a screening, “difficulty when doing the exams in a big hospital,” and “traffic/transportation.” Interestingly, “radiation exposure” was the least frequently identified barrier (1%) in the study sample.

### 3.4. Factors Predicting BCS Behavior and Intention

Logistic regression analyses were conducted modeling the odds of ever having screening done within the previous five years. The *R* ^2^ of Cox and Snell and Nagelkerke showed that 14%–21% of variance of BCS practice was explained by the selected variables, and the model had an overall correct prediction of 71%. Among variables included in the model, demographic variables (age, education, and insurance) did not have significant prediction with screening behavior while increased self-efficacy; having performed monthly self-exams and having had clinical breast exams in the past two years were significant predictors of having had a mammogram or an ultrasound in the previous five years. Among these variables, for those who reported having recent clinical breast exams done in the past two years, those who performed monthly breast self-exams and those who reported higher self-efficacy were more likely to get the mammogram or ultrasound done in the past five years (odds ratios = 4.5, 3.0, and 1.9 resp., see Table 4). Interestingly, the interaction between recent CBE performance and monthly breast exams had an odds ratio less than one, meaning those women who have performed both exams more frequently report they were less likely to get mammograms or ultrasounds. In terms of factors associated to the participants' intention to obtain mammograms in the future, the results from Pearson's correlation showed that only one variable, knowledge, is associated with women's intention for getting screening in the next year (*r* = 0.15, *P* < 0.01), that is, women with higher knowledge scores were more likely to indicate that they plan to get screening in the future. 

## 4. Discussion

While breast cancer incidence has been historically less prevalent in Asian countries compared to the West, recent statistic data reveal breast cancer incidence rates have had a 20–30% increase in China in the past decade according to China's urban cancer registries [25]. To our knowledge, this is the first study that investigated practices and knowledge toward breast cancer screening among Chinese women outside of Beijing and Shanghai. The current study surveyed women participants from Wuhan, a metropolitan area located in the heart of China, which has developed very quickly and whose economy has kept a continual and steady growth. China became a majority urban country in 2012 and Wuhan is among China's fastest-growing city in population and home to significant economic activity. Similar to Shanghai, Wuhan has a large increase in population with more than nine million citizens. 

The results provided evidence that few Chinese women in the study sample participated in any BCS modalities (mammography, ultrasound, CBE, and/or breast self-exams); specifically, only 30% of women reported they had CBE done in the past two years and 70% of women never had a mammogram. The rates reported in the current study were even lower than the rates of (43–64%) in previous studies conducted in other regions of China (i.e., Beijing, Shanghai, Guangzhou, and Xi'an) [26, 27] and in other studies on Asian American women (including Chinese American) [23, 28]; the low rates of breast cancer screening reported in the current study can be associated with lack of awareness of breast cancer, possessing high misconceptions toward early detection, lack of access to screening programs, and social and economic barriers.

Nevertheless, the mediating effects of psychosocial and cultural variables on the impact of breast cancer intervention in low-to-middle income countries (LMC) are still understudied [29]. The personal presentations of illness influence women's responses to prevention and screening campaigns as well as the likelihood of initiating and complying with recommended guidelines in breast care and screening. Public education that includes messages conveying the idea that breast cancer is curable in the majority of women when it is detected early and followed with timely and proper treatment is a key first step for successful breast health programs [30]. 

In terms of knowledge and attitudes related to breast cancer screening, Chinese women participants in the current study had much lower knowledge levels about breast cancer and risk factors, perceived less susceptibility, and reported greater barriers to screening and lower self-efficacy compared to study findings on immigrant Chinese women in the U.S. [28] The study utilized both qualitative and quantitative methodology that provided less research-derived and broader understanding regarding barriers to breast cancer screening. With open-ended questions, women were able to provide in their own words why they did not perform BC screening. Interestingly, the most prevalent theme is low priority, followed by the theme of “Feeling OK.” More than half of the women felt that breast cancer screening was not necessary because they did not experience any symptoms or abnormality. The findings were consistent with other studies carried out in Korea [31, 32], Singapore [33, 34], and Malaysia [35], which showed that the women did not perceive the importance of early detection of breast cancer. Similarly to the findings reported from Im's study (2004) for Korean women [32], study participants in China did not perceive the need for breast cancer screening if they did not have any symptoms or family history, because they thought their risk of developing breast cancer was low. 

The study participants were also cognizant about their lacking awareness in breast cancer. The data lent support for the recent release of the Consensus Statement from the Breast Health Global Initiative that indicated a lack of public awareness in the importance of early detection of breast cancer in LMCs [30]. Identifying barriers to early detection is a critical component of breast cancer control programs and developing effective strategies to overcome barriers should involve both community and public health leaders [30]. Practical evidence-based strategies are needed for effective communication with the public in promoting early detection of breast cancer [30]. In the current study, the results revealed several areas of misconceptions or incorrect information perceived by study participants. For example, participants underestimate the importance of regular screening when they provide responses such as they were too old; therefore, screening was not necessary, or they had done self-exams so they do not need additional mammograms. Campaigns can be most effective if they are carefully targeted to meet the unique needs of the targeted audience in LMCs [36–38]. Messages can be designed to appeal to these identified misconceptions with culturally acceptable languages and channels, for example, via health promoters or lay health advisors who have developed trusted relationships with targeted at-risk women in the community in low-resource countries. In addition, self-efficacy emerged as a significant predictor suggesting that the intervention content was successful in addressing this key concept by offering effective strategies to overcome their personal barriers and increase confidence in obtaining regular screening.

The study found positive associations between ever having had a mammogram, having CBE within the past two years, and performing monthly breast-exams, that is, adoption of one screening practice was positively correlated with other breast screening modalities in the current study. This finding was consistent with a previous study conducted in the U.S. among immigrant Asian women [39]. While CBE may be the more commonly accessible screening in China, the encounter can act as an ideal educational moment for care providers to educate women about the importance of mammography and improve adherence with both screening modalities. It is also equally important that during these educational encounters the need to emphasize the importance and relevance of screening, particularly among previously never-screened women, and to encourage women who had previously performed CBE and breast self-exams (BSEs) that mammogram screening cannot be replaced by the compliance of regular CBEs and monthly BSEs.

 Financial cost emerged as one of the top five barriers for Chinese women in this study to access mammography screening. This finding is consistent with other studies done in Hong Kong and immigrant Chinese women in the U.S. Women's lack of resources, combined with cultural and attitudinal barriers, along with limited access to affordable mammography services directly affect the likelihood of BCS since women with low incomes may have many other competing priorities related to survival that make breast cancer screening a low priority. The responses of study participants suggest that they might be amenable to engage in more BCS if they were more aware of what was available to them and if they still have access to preventive services such as mammograms after they retire. In 2008, the Chinese Ministry of Health started a program on early detection and treatment of cancer sponsored by central financial assistance that provide CBE, mammography, and ultrasound for women in 30 provinces in China. Efforts to develop programs and/or policies to improve breast cancer screening will be more successful if the implementation includes strategies to increase cancer knowledge, awareness, and information and complementary efforts to help women address emotional, socioeconomic, and other barriers to clinical breast exam and mammography use.

The current study had several limitations. First, due to the nature of self-report data, there may have been over- or under-reporting of screening practices. Future studies with a combination of self-report and verification from objective chart reviews are warranted to ensure the accuracy in reporting socially desired behaviors, such as cancer screening. Second, because the use of convenience sample and small sample size, this approach can lead to sampling error; therefore, the results cannot be generalized to other regions/provinces in China, a country with more than 1.3 billion citizens with diverse socioeconomic and cultural backgrounds. Nevertheless, based on the demographics in the current study sample, the study recruited relatively diverse groups of women with a wide range of income and educational levels. 

## 5. Conclusions

 Changes in breast cancer incidence and distribution of breast cancer patterns in Chinese women present opportunities for developing and implementing effective public health programs to promote BCS for women in China. It is essential that public health campaigns focus on the importance of early detection and prevention that target both general public and health care professionals working directly with their clients. The findings of this descriptive correlational study provide a foundation to better understand beliefs and attitudes of Chinese women toward BCS. The concept of screening while asymptomatic is lacking and a low priority toward BCS was observed in the current study. As a result, this study highlights a critical need among general public, health professionals, and the health care system to work collaboratively toward narrowing the gap in BCS and to improve the quality of breast cancer care in this population. 

## Figures and Tables

**Table 1:** Demographic characteristics of study participants.

Characteristic	Frequency	%
Age, years		
40–49	155	39
50–59	179	45
60+	65	16
Marital Status		
Married	385	96
Widowed/divorced/separated	14	4
Never married	1	—
Income (monthly)		
<$2600 RMT (equals to U.S. $410)	77	20
2601–4600 RMT (U.S. $411–$725)	189	47
4601–11500 RMT (U.S. $726–$1,812)	107	27
11501–16500 RMT (U.S. $1,813–$2,600)	19	4
16501–23000 RMT (U.S. $2,601–$3,625)	4	1
>23001 RMT (>U.S. $3,626)	4	1
Occupation type		
Unemployed	43	11
Blue collar	90	23
White collar	113	28
Retired	148	37
Other	4	1
Level of education		
None	16	4
Elementary school	23	6
Middle school	97	24
High school	179	45
Vocational school	32	8
Bachelor's degree	45	11
Master's degree	7	2
Diagnosis of breast cancer		
Yes	8	2
No	391	98
Do not know	1	0
Family history of breast cancer		
Yes	8	2
No	391	98
Health insurance		
Yes	189	47
No	211	53
Health insurance covering mammograms (*n* = 189)		
Yes	74	35
No	35	17
Do not know	102	48

RMT: Renminbi, Chinese currency.

Note:

(1) due to missing data, the frequency did not add up to the total sample size.

(2) marital status and education level distribution is similar to China 2000 census; however, the education level in this sample is higher. The comparisons in other variables were not available.

**Table 2:** Knowledge beliefs and practice of breast cancer screening.

Characteristic	Frequency	%
*Breast self-exams *		
Heard of breast self-exam (*n* = 400)		
No	79	19.8
Yes	321	80.2
Practice of breast self-exam (*n* = 348)		
Never	166	47.7
Once every year	1	—
2–6 times a year	93	26.7
7–11 times a year	20	5.8
Once every month	68	19.5
*Clinical breast exams (CBE) *		
Heard of CBE (*n* = 398)		
Yes	132	33.2
Obtaining most recent CBE (*n* = 398)		
Never	141	35.2
Do not remember	44	11.0
More than 2 years ago	80	20.0
Less than 2 years	135	33.8
*Mammograms *		
Heard of mammograms (*n* = 400)		
Yes	165	41.2
*Obtaining mammogram(s) in past 5 years (*n* = 326)		
Yes	83	24.5
Obtaining mammogram(s) in the past year (*n* = 399)		
Yes	52	13.0
*Ultrasounds *		
Heard of ultrasounds (*n* = 399)		
Yes	191	47.9
*Obtaining ultrasound(s) in past 5 years (*n* = 326)		
Yes	77	23.6
Obtaining ultrasound(s) in the past year (*n* = 398)		
Yes	48	12.1

Beliefs: CMSBQ Subscales	Mean	S.D.

Self-efficacy (range: 1–4)	2.4	0.6
Barriers (range: 1–4)	2.5	0.4
Benefits (range: 1–4)	3.2	0.5

Note: due to missing data, the frequency did not add up to the total sample size.

*The calculation of this item was based on a selective sample of 326 women ages between 45 and 75.

**Table 3:** Self-reported barriers toward breast cancer screening.

Characteristic	Frequency	%
Low priority	217	63
Feeling OK	202	58
Lack of info/knowledge	145	42
Cost	114	33
Logistics	34	10
Had other exam(s) earlier	26	8
Fear of finding cancer	19	6
Discomfort	16	5
Doctors not recommending it	9	3
Radiation	4	1

**Table 4:** Factors associated with breast cancer screening.

Variables	Coefficient	Standard error	Significance *P*	Odds ratio	95% CI
Age	0.00	0.02	0.81	1.00	(0.97, 1.04)
Education	0.14	0.13	0.29	1.15	(0.89, 1.49)
Insurance	0.25	0.28	0.37	1.28	(0.75, 2.21)
Perform BSE	1.51	0.43	0.00**	4.53	(1.94, 10.57)
Perform CBE	1.11	0.34	0.00**	3.04	(1.56, 5.92)
Self-efficacy	0.67	0.31	0.03*	1.95	(1.07, 3.55)
Barriers	0.16	0.41	0.67	1.18	(0.52, 2.64)
Benefits	−0.05	0.30	0.88	0.95	(0.53, 1.71)
Knowledge	−0.07	0.09	0.45	0.93	(0.78, 1.12)
Interaction between CBE and BSE	−1.26	0.63	0.04*	0.28	(0.08, 0.97)

Note: BSE: breast self-exams, CBE: clinical breast exams; **P < 0.01, *P < 0.05.
